# Caregiver perceptions of in-home COVID-19 testing for children with medical complexity: a qualitative study

**DOI:** 10.1186/s12887-022-03550-5

**Published:** 2022-09-08

**Authors:** Anna Jolliff, Nicole E. Werner, Hanna J. Barton, Kristina Devi Howell, Michelle M. Kelly, Makenzie Morgen, Mary Ehlenbach, Gemma Warner, Barbara Katz, Madeline Kieren, Gregory DeMuri, Ryan J. Coller

**Affiliations:** 1 Department of Health and Wellness Design, Indiana University School of Public Health-Bloomington, 1025 E 7th St, Bloomington, IN 47405 USA; 2 Department of Industrial and Systems Engineering, University of Wisconsin – Madison, 1550 Engineering Drive, Madison, WI 53706 USA; 3 Department of Pediatrics, School of Medicine and Public Health, University of Wisconsin-Madison, Madison, USA; 4 Family Voices of Wisconsin, Madison, WI USA

**Keywords:** Children with medical complexity, Direct antigen rapid testing, COVID-19

## Abstract

**Background:**

In-home direct antigen rapid testing (DART) plays a major role in COVID-19 mitigation and policy. However, perceptions of DART within high-risk, intellectually impaired child populations are unknown. This lack of research could negatively influence DART uptake and utility among those who stand to benefit most from DART. The purpose of this study was to describe caregivers’ perceptions of an in-home COVID-19 DART regimen in children with medical complexity, including the benefits and limitations of DART use.

**Methods:**

This qualitative study was a subproject of the NIH Rapid Acceleration of Diagnostics Underserved Populations research program at the University of Wisconsin. We combined survey data and the thematic analysis of semi-structured interview data to understand caregivers’ perceptions of in-home COVID-19 testing and motivators to perform testing. Caregivers of children with medical complexity were recruited from the Pediatric Complex Care Program at the University of Wisconsin (PCCP). Data were collected between May and August 2021.

**Results:**

Among *n* = 20 caregivers, 16/20 (80%) of their children had neurologic conditions and 12/20 (60%) used home oxygen. Survey data revealed that the largest caregiver motivators to test their child were to get early treatment if positive (18/20 [90%] of respondents agreed) and to let the child’s school know if the child was safe to attend (17/20 [85%] agreed). Demotivators to testing included that the child could still get COVID-19 later (7/20 [35%] agreed), and the need for officials to reach out to close contacts (6/20 [30%] agreed). From interview data, four overarching themes described perceptions of in-home COVID-19 testing: Caregivers perceived DART on a spectrum of 1) benign to traumatic and 2) simple to complex. Caregivers varied in the 3) extent to which DART contributed to their peace of mind and 4) implications of test results for their child.

**Conclusions:**

Although participants often described DART as easy to administer and contributing to peace of mind, they also faced critical challenges and limitations using DART. Future research should investigate how to minimize the complexity of DART within high-risk populations, while leveraging DART to facilitate safe school attendance for children with medical complexity and reduce caregiver burden.

**Supplementary Information:**

The online version contains supplementary material available at 10.1186/s12887-022-03550-5.

## Introduction

In-home direct antigen rapid testing, or DART, now plays a major role in COVID-19 mitigation and policy [1, 2]. In December 2021, the US government shared a plan to distribute 500 million direct antigen rapid tests in an effort to slow the spread of COVID-19 [3]. While emerging research suggests that DART is acceptable among US adult populations [4], perceptions of using DART at home within high-risk child populations are unknown. The lack of research in this area could negatively influence the uptake or utility of DART. The present study examines the use of DART within one model high-risk population: children with medical complexity, who represent ~ 0.5–5% of the U.S. child population and are defined as children with multiple severe chronic conditions, significant functional limitations, and high service needs [5, 6].

Individuals with medical complexity are at higher risk for contracting COVID-19, and, if infected, are more vulnerable to severe symptoms, hospitalization, and death [7–9]. In the absence of testing, clinicians and caregivers of children with medical complexity may be slower to suspect COVID-19 in their children, whose baseline health may always include symptoms consistent with COVID-19 (e.g., cough, variable vital signs, oxygen needs) [10]. Further, children with medical complexity often face neurocognitive impairments and can have difficulty communicating about their symptoms [11]. Thus, access to consistent COVID-19 testing for children with medical complexity is paramount to facilitate the rapid detection of COVID-19, earlier monitoring, use of existing clinical action plans (e.g., for respiratory illness), and consideration of COVID-19-directed therapies. Rapid awareness of COVID-19 status could also benefit families of children with medical complexity by ensuring appropriate precautions are taken by other caregivers assisting the child in the event of a positive result, and reducing unnecessary school or work absences in the event of a negative result.

The objective of this study was to describe caregivers’ perceptions of an in-home COVID-19 DART regimen for children with medical complexity, including the benefits and limitations of DART use. Findings from this study may be used to inform policy, promote uptake, and understand under- or over-use around DART within high-risk populations.

## Methods

### Setting, sample, and design

This qualitative study was a subproject of Aim 1 of the NIH Rapid Acceleration of Diagnostics (RADx) Underserved Populations (RADx-UP) research program at the University of Wisconsin [12]. Aim 1 was to evaluate feasibility of in-home DART use among a cohort of family caregivers and their children with medical complexity recruited from the Pediatric Complex Care Program (PCCP). Interview participants were family caregivers who were screened by phone for the following eligibility criteria: caregivers were English-speaking, resided in Wisconsin, and had access to a web-enabled device to complete the interview. Their children with medical complexity were between the ages 5–17 and attended in-person school prior to the pandemic. Children with medical complexity in the PCCP have ten or more annual clinic visits (or five or more annual hospital days), see three or more specialists, and have three or more organ systems affected by chronic disease. When participants provided informed consent to the feasibility study, they also provided informed consent for the subproject. Children with medical complexity were not considered participants in this subproject as they were not interviewed. Participants were informed that they might be approached for an optional interview, which they were free to decline without adversely affecting their participation in the larger study. Two research assistants reached out to larger study participants by phone to inquire about participation in the subproject. The first 20 to enroll in the interview portion were accepted, based on previous research suggesting that a sample size of 20 is sufficient to obtain thematic saturation [13, 14]. After enrollment, no participants dropped out of the study.

Study team members trained caregiver participants to administer the BinaxNOW Rapid Antigen Self-Test. The BinaxNOW Rapid Antigen Self-Test is a point-of-care lateral flow immunoassay used for the qualitative detection of a protein antigen from SARS-CoV-2 via nasal swab. Caregivers administered the BinaxNOW test twice weekly during a 3-month surveillance period from 5/2021–8/2021.

### Procedure

We used semi-structured interviews and a survey to understand caregivers’ perceptions of in-home COVID-19 testing. The survey instrument was created using National Institutes of Health (NIH) RADx-UP (Rapid Acceleration of Diagnostic – Underserved Populations) Tier 1 and selected Tier 2 Common Data Elements (https://radx-up.org/research/cdes) adapted for the pediatric population [15]. The survey included standardized questions about demographics, housing, employment, health status, and perceptions around COVID-19 school safety, testing, and vaccination, and the present study reports on the demographics and testing perceptions questions. All feasibility study participants completed the survey, which was self-administered using REDCap web-based survey software [16, 17] . A research team with expertise in qualitative research, pediatric complex care, and human factors engineering developed the interview guide (NW, RC, HB, MM). The research team also included parent partners, who helped to ensure that the interview guide was informed by lived experience with caring for children with medical complexity. Questions in the interview guide were based in part on the health belief model, which cites perceived barriers and perceived benefits to a health-promoting behavior (in this case, a DART regimen) as strong predictors of whether a person will engage in that behavior [18]. The interview guide was also based on our research questions, which meant to address a gap in the literature on caregivers’ perceptions and experience of using a DART regimen with their children with medical complexity. After completing five interviews, the order in which questions were asked was changed to facilitate rapport building and to create a more efficient interview. See the supplementary file for the final interview guide. After the interview was complete, participants were asked to complete a survey on motivations for testing for COVID-19. All participants provided informed consent prior to completing the interview and survey and were told that their data would be de-identified prior to inclusion in any resultant publications. This study was approved by the university institutional review board.

Interviews were 60-minutes in duration and conducted with video conferencing software. Only two researchers were present; one researcher conducted the interview (HB, graduate student in human factors engineering) while another took field notes (MM, medical student). Participants had previously met the interviewer during recruitment but had no further familiarity. Interviews were audio and video recorded, transcribed verbatim, and transferred to Dedoose 9.0.17 qualitative analysis software for analysis [19]. Participant numbers were randomly assigned. All interviews and surveys were completed in July or August of 2021.

### Data analysis

Survey data were summarized with descriptive statistics. We conducted a team-based thematic analysis in which the full, interdisciplinary research team contributed to the thematic coding framework [20, 21]. We focused our analyses on identifying perceptions of in-home testing among caregivers of children with medical complexity, with an emphasis on the benefits and limitations to in-home DART use. One researcher (AJ) performed structural coding guided by the research objective to identify initial themes. Those themes were applied to 10% of the transcripts by two other research team members during a team analysis meeting (HB and NW). Themes were revised during that session and then brought to the entire research team for discussion. Themes were further revised and applied by AJ in a second round of coding, with coding progress discussed through regular meetings with the research team. Last, themes were iteratively refined in the process of manuscript development with the whole research team. Qualitative data are reported according to the COREQ checklist.

## Results

### Participants

Among the *n* = 20 caregivers of children with medical complexity, children ranged in age from 5 to 16 years (M = 10.1), and 11/20 children (55%) were assigned male at birth. Children had a median (interquartile range, IQR) of 7 (5–9) subspecialists and 12 (7–14) scheduled daily medications. Within the sample, 16/20 children (80%) had a neurologic chronic condition and 12/20 (60%) used home oxygen. Additional demographic and clinical data are summarized in Table 1.

**Table 1:** Caregiver and child demographic and clinical data

Demographic variable	N (%)	
	Caregiver	Child
Gender		
Female	18 (90)	9 (40)
Male	2 (10)	11 (55)
Prefer not to say	0 (0)	1 (5)
Ethnicity		
Non-Hispanic	18 (90)	17 (85)
Hispanic	1 (5)	2 (10)
Prefer not to say	1 (5)	1 (5)
Race		
White	16 (80)	13 (65)
Asian	2 (10)	1 (5)
Native Hawaiian or Pacific Islander	0 (0)	1 (5)
American Indian or Alaska Native	0 (0)	1 (5)
Multiple races	0 (0)	4 (20)
Prefer not to say	2 (10)	1 (5)
Selected Child Clinical Characteristics		
Neurologic chronic condition		16 (80)
Cardiovascular chronic condition		9 (45)
Genetic/metabolic chronic condition		7 (35)
Enteral tube		17 (85)
Home oxygen	12 (60)	
Tracheostomy	4 (20)	

For race and clinical characteristics, respondents could select more than one response option

### Testing motivations survey

**Fig. 1:**
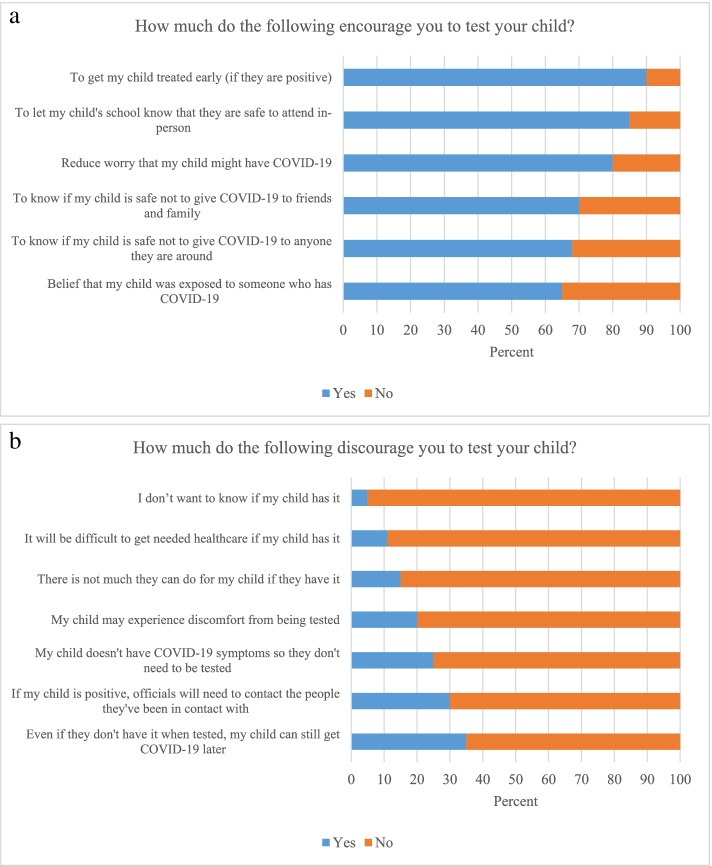
**a** Motivators to COVID-19 testing for Caregivers of Children with Medical Complexity. **b** Demotivators to COVID-19 testing for Caregivers of Children with Medical Complexity

The largest caregiver motivators to test their child were to get early treatment if positive (18/20 [90%] of respondents agreed) and to let the child’s school know if the child was safe to attend (17/20 [85%] agreed). Demotivators to testing included that the child could still get COVID-19 later (7/20 [35%] agreed that this discouraged testing), and the need for officials to reach out to close contacts (6/20 [30%] agreed). Full survey results on caregiver motivators and demotivators can be found in Fig. 1a and b, respectively.

### Caregiver interviews

Caregivers of children with medical complexity varied in their perception of the benefits and limitations of in-home COVID-19 testing. Four overarching themes related to perceptions of in-home COVID-19 testing were identified. Caregivers perceived testing on a spectrum of benign to traumatic and simple to complex. Caregivers varied in the extent to which testing contributed to their peace of mind and the implications of test results for their child. See Table 2 for quotes representing each theme and subtheme.

**Table 2:** Representative quotes from caregivers on benefits and limitations of in-home COVID-19 testing

Theme	Subtheme	Quote	Representative quotes (Participant #)
Testing ranges from benign to traumatic		Q1	This is like a walk in the park compared to other things she needs. … It’s such a brief twirl on each side that it’s over before it’s even really begun (P21).
		Q2	So I think the first time was very challenging because he does have a very heightened anxiety level with any sort of procedure … Kids that have been poked and everything, you know, they get really apprehensive about anything coming close to their body (P29).
		Q3	The first few times I was like, oh, man, I might not be able to do this to [name] because he’s screaming, and I don’t want to traumatize him twice a week. But I think he’s learned too, like I said, it doesn’t go straight up his nose (P10).
Testing ranges from simple to complex	Perceived ease of testing procedure	Q4	You know, the testing itself is not difficult. I think the process is pretty simple. So, you know, just having fewer steps really made it less complicated and easier to administer (P29).
		Q5	I guess it’s a lot of things to try and keep next to each other. So it’s like I want to make sure that everything is open and still good to go, and I think the only issue I have is doing the six drops while holding the Q-tip in my hand so it doesn’t touch anything, because I don’t think I was supposed to do it before I, before that swab goes into the nose, because you don’t want them drops sitting there …. I mean, I just wish it was all very simple to where you just swab it and then, I don’t know, throw it in a bag, and if it turns this color, it’s negative, or if it turns that one, it’s positive (P22).
	Need for assistance with testing	Q6	We typically try and do it together. So it’s, we have two adults instead of, like the first time we did it, I just did it by myself, and we learned that that probably wasn’t the best thing. I think he does a really good job of making sure that it’s swabbed enough, whereas if I were doing it with her reaction, I don’t think that I would do as good of a job as he would to make sure that I would swab as well as I probably should (P13).
	Difficulty of accessing testing	Q7	I think that every insurance should pay for it for in home. If you’re going to make a big deal about this COVID stuff, then you need to give people stuff to be able to deal with it. And it should be automatically put in homes for people (P20).
		Q8	I saw [direct antigen rapid tests] in the stores, right? … I was mildly horrified at how expensive they are … I’m a little bit disappointed that they’re like $20 or $25 or whatever for a box of two, right? … I would want people to not be discouraged, right? … I think that would deter people. It would deter me. I wouldn’t do it (P24).
		Q9	Yeah, like a much more affordable option if it comes through to just go pick up a kit if your kid is sick or something like that and you want to test them for a certain, like a COVID-19. This, to me, seems like a way better option than going to all the testing facilities that used to be and, or even into the hospital, because, I mean, obviously there’s huge costs sometimes associated with that, and not everybody is going to be able to do that (P13).
Testing contributes to peace of mind		Q10	[The tests] are fast. I like that they’re same day. I know a lot of times, like I know the last time she was really sick, we ended up quarantining for two weeks, and the swab she got took like 72 hours or more to result, and we didn’t know what we were dealing with, and we didn’t know if we should go in or how bad it was going to get, because we didn’t know what she had at that point. So it was really hard to wait those three days to find out that it was negative in the end and it wasn’t what we were dealing with. But it made it a lot more scary to wait that long, thinking that could be what it was. So the immediate reassurance that she’s negative is, means a lot (P14).
	I would feel better if everyone were tested	Q11	Can we have like the whole school get tested? That would make me feel way more confident. I would say that’s the big thing. I’m like, well, I can say for my kid, but like that doesn’t help the people around him” (P17).
	Vaccination is more protective than testing	Q12	I prefer that everybody would be vaccinated around her. … I can’t say, hey, are you vaccinated? You know, I only want vaccinated people around her. And then as far as the kids, I don’t know how that looks like either. So even with my testing, like I’m happy that I have it in case that I need it when she, well, I probably will be testing her a lot more frequently if she goes to school (P22).
Test results have implications for my child		Q13	I do wonder sometimes if I’m giving myself a false sense of security knowing that in the school district I’m in … if we send them home quarantined because of symptoms, they’re not allowed back in school without the [PCR] testing, right, the other kind of test. We don’t accept this test because it’s not as reliable. So there’s a part to me that’s like am I putting all my eggs in one basket to say this is the end all be all? But at least it is a tool. … I know that there’s [PCR] testing out there that’s more accurate, theoretically, or that’s what I’ve been told, so that just sits in the back of my mind … But it’s better than nothing, so (P18).
		Q14	But then it was frustrating, because her school wouldn’t take these test results as an okay for her to go back to school. So we then had to take her to the hospital to test her (P28).

#### Theme 1: testing ranges from benign to traumatic

Within the testing procedure, caregivers perceived the sample collection component in particular as ranging from benign to traumatic. Caregivers who experienced sample collection as benign tended to cite the relative non-invasiveness of the swab and the quick recovery time of their child. Although they acknowledged that their child was “not super thrilled” (Participant 8) to be swabbed, and that it was “not the most comfortable thing in the world” (P29), some participants noted that their child would “recover from it really quickly” (P30) and then be “good to go” (P4). One caregiver shared that, compared to other medical procedures their child experienced, sample collection for COVID-19 testing seemed benign (see Table 2, Quote 1).

In contrast, caregivers who experienced sample collection as more traumatic said that their child would become “upset” (P30) or “nervous” (P26) at the prospect of testing and would often fight their attempts to obtain a sample. One caregiver explained that the nasal swab seemed to call up memories of medical trauma (Quote 2). Several participants explained that their child had previously undergone the more invasive PCR testing experience with a deep nasal swab. As one caregiver explained, this caused the child to be more reactive to even shallow swabbing (Quote 3).

#### Theme 2: testing ranges from simple to complex

Families varied in the extent to which they perceived in-home testing as simple versus complex. Variables that contributed to complexity included the procedure itself, the degree to which assistance was required for testing, and caregivers’ ability to access and pay for testing.

#### Theme 2, subtheme 1: perceived ease of testing procedure

Most caregivers of children with medical complexity tended to describe the testing procedure itself as simple. Caregivers described the procedure as “clear,” (P30) “simple” (P29) and “easy” (P13, P18, P4, P24, P14, P21). One caregiver noted that administering and interpreting the test required only a few straightforward and easy-to-memorize steps (Quote 4). However, not all caregivers perceived the testing steps as simple. One caregiver experienced in-home testing as difficult to perform correctly and could imagine an easier process (Quote 5).

#### Theme 2, subtheme 2: need for assistance with testing

Caregivers varied in the extent to which they required the support of others to administer in-home tests. Many caregivers were the only member of the household who performed the test, reasoning that their child was often “more comfortable” (P26) or “more compliant” (P10) with them than with anyone else. However, a subset of caregivers required the collaboration of others to perform the test, which added complexity to the process. One caregiver explained why it was vital that two caregivers were present for to the testing process, indicating that she doubted her ability to capture an adequate sample (Quote 6).

#### Theme 2, subtheme 3: difficulty of accessing testing

Caregivers identified access to testing as a third contributor to complexity. For caregivers involved in the study, tests were provided so that access was a non-issue. However, some participants lamented the relative inaccessibility of testing for those not involved in the study and therefore not receiving free test kits. One participant wished that “all of the parents [of children] with special needs in the state could participate” (P10). Another caregiver felt that, given the public health priority of containing COVID-19, insurance companies should cover the cost of testing (Quote 7). One caregiver added that, if test kits were not provided free of charge, she would not do in-home testing at all (Quote 8).

For other caregivers, living far from testing facilities or hospitals meant in-home testing was *more* accessible by comparison. These caregivers were able to dodge the considerable time and complexity associated with transporting their child. One following caregiver perceived in-home testing as a less expensive and more affordable option for families than going to the hospital or testing facilities (Quote 9).

#### Theme 3: testing contributes to peace of mind

The consensus among caregivers was that access to in-home testing increased peace of mind. Caregivers described feeling “more control” (P29), a sense of “security” (P13), feeling more “comfortable” (P9), and feeling “safer” with the tests on-hand (P21) – particularly in comparison with getting tested at the doctor’s office and “walking into a germ fest” (P20). Participants also lauded the “quickness” (P28) of the tests, which enabled them to “catch [COVID-19] right away” (P4) and be more “reactive” (P8). One participant described how emotionally challenging it was to wait 3 days for test results; in contrast, the fast results of in-home tests contributed greatly to peace of mind (Quote 10).

#### Theme 3, subtheme 1: I would feel better if everyone were tested

Caregivers were often just as concerned about limiting exposure to COVID-19 as they were about determining the child’s COVID-19 status. Accordingly, it was common for caregivers to use in-home tests to test people in the child’s network. This included other household members (e.g., stepdad, brother, and the caregivers themselves) as well as those outside the home (e.g., nanny, grandma, and a neighbor child) (P24, P28, P29, P17, P5, P21). However, caregivers sometimes noted that there were others (e.g., child’s classmates or teachers) they would like to test for their own peace of mind, but could not (Quote 11).

#### Theme 3, subtheme 2: vaccination is more protective than testing

At the time of interviews, vaccinations were not available for children under 12 years of age. However, some caregivers spontaneously mentioned the role of vaccination, saying that this was ultimately more central to their peace of mind than early detection of COVID-19. One caregiver described testing her child as the second-best option to asking her child’s care team and teachers about their vaccination status (Quote 12).

#### Theme 4: test results have implications for my child

Participants varied in the degree to which they perceived the results of in-home tests as having important and actionable implications for their child. Some caregivers perceived the implications of test results to be limited because, compared to the RT-PCR test, DART results were “not always like maybe as accurate” (P4). Another caregiver worried that negative test results provided a false sense of security (Quote 13).

The results of COVID-19 tests also had limited impact on whether children with medical complexity could attend schools. Participants’ schools typically did not accept DART results as evidence that the child was COVID-negative. This was especially problematic for participants whose children often displayed COVID-like symptoms (e.g., runny nose, cough, difficulty breathing) due to their medical condition, but who had, nonetheless, obtained negative test results. One participant had to pick her son up from school “over 27 times” because he was displaying symptoms typical of his medical condition, despite having obtained repeated negative test results (P10). Another participant concurred that their child’s school did not accept in-home tests as evidence that the child was safe to return (Quote 14).

## Discussion

This study is among the first to examine perceptions of in-home COVID-19 testing using direct antigen rapid tests within an at-risk population. Although typically described as fast and easy, our results underscore that, especially within high-risk and neurocognitively affected populations, some may face critical challenges and limitations when using DART. These findings should influence communication and empathy between healthcare professionals and caregivers of children with medical complexity. Further, given the recent federal investment in in-home DART [3], these findings may inform public health messaging around DART use to increase detection and slow the spread of COVID-19.

Our findings are consistent with existing research showing variation in caregiver perceptions of performing healthcare procedures at home for children with medical complexity. For example, a study by Desai and colleagues (2016) found that some caregivers of children with medical complexity managing hospital to home transitions felt confused and insecure about home care tasks, while others felt confident and prepared [22]. Blackmer and colleagues (2020) found that parents of children with medical complexity vary in their confidence around medication management, with nearly 20% of participants unsure of medication side effects and unsure how to respond to those side effects if they should occur [23]. Although research on COVID-19 testing among children with medical complexity is limited, our findings align with existing research on COVID-19 testing among healthy adults, which has shown that invasiveness, ease of administration, and cost of testing contribute to more positive or negative perceptions [24]. Likewise, similar to previous research on healthy adult subjects [4], the present study showed that the use of DART provided most participants with peace of mind. Public health leaders wishing to increase uptake of DART are advised to highlight their relatively non-invasive nature and ability to improve peace of mind. Further, it is imperative that policymakers work to make DART inexpensive or free, especially for medically complex individuals who require access to many tests to create a testing regimen.

Our results illustrated that administering direct antigen rapid tests was a simple process for many, but not all, caregivers. This is consistent with previous research on German adults, which found that 81% considered DART easy to perform [25]. However, some caregivers in this study perceived the use of DART as more complex and even traumatic. Particularly for those children with medical complexity who had previously undergone the deeper nasopharyngeal swabbing, even the relatively shallow nasal swab testing associated with BinaxNOW seemed to elicit anxiety. This is consistent with previous research showing that those with a history of medical trauma may experience arousal and avoidance when faced with a trigger [26]. When introducing a DART regimen to caregivers of children with medical complexity, therefore, parents and clinicians should acknowledge, empathize with, and help troubleshoot strategies to avoid the elicitation of a trauma response. Test kit designs, instructions, and public health messaging must account for the important subsets of high-risk individuals, including those with cognitive impairment, who may struggle to perform DART at home.

Our thematic analysis results also showed that in-home testing, and particularly the speed at which results could be obtained, improved caregivers’ peace of mind. Survey data on motivators for testing similarly suggested that speedy results may promote peace of mind by ensuring earlier access to treatment if test results are positive. While the survey results suggested that caregivers were motivated by a potentially quicker return if results are negative, this differed from the perspective expressed during interviews. Interview data showed that prevailing school policies did not always allow negative DART results to clear children for school attendance. In their survey responses, participants acknowledged that even if test results are negative, their children could still contract COVID-19 later. Interview results showed that the COVID-19 and vaccination status of *other* individuals in the child’s environment was harder to enforce, yet often contributed more to caregivers’ peace of mind. Some caregivers and schools perceived DART as lacking validity, which could undermine clinical and public health strategies promoting DART as an important component. Thus, public health messaging should highlight the sensitivity of a DART regimen, which is affected by variables like access to multiple tests and quick identification of infected individuals [27]. Future research should investigate how to safely leverage the results of antigen tests in order to facilitate safe school attendance for children with medical complexity and reduce caregiver burden.

### Limitations

Several limitations of this study should be considered. The sample of caregivers was disproportionately white and female and findings may not generalize to other populations. We did not elicit data to describe the extent of additional caregiver supports and whether this influenced testing procedures or perceptions (for example, if a caregiver had assistance when testing). Further, this study did not examine perceptions of children with medical complexity, but rather the perceptions of their caregivers. Caregivers had been specially trained by study staff to administer DARTs; thus, our finding that many caregivers found the tests simple to administer may not be generalizable to caregivers without this training. Finally, this study was conducted prior to the approval of vaccinations of children between 5 and 12 years of age. Future research should examine how perceptions of in-home testing changed with the approval of the COVID-19 vaccine for children.

## Conclusions

The present study sheds light on perceptions of direct antigen rapid testing among caregivers of children with medical complexity. In order for policymakers and medical professionals to promote the use of in-home rapid tests as part of a broader COVID-19 mitigation plan, it is vital to first understand and then react to the perceptions of end users in high-risk populations. This will lead to an empathetic stance from which we can promote test uptake, minimize caregiver burden, correct misconceptions, and achieve the goal of slowing the spread of COVID-19.

## Supplementary Information

**Additional file 1.** Semi-Structured Interview Guide.

**Additional file 2.** COREQ (COnsolidated criteria for REporting Qualitative research) Checklist.

## Data Availability

The datasets generated and/or analysed during the current study have not been deposited into an open access data repository due to the nature of the data. They are transcripts of personal dialogue, have unique structural and technical challenges for storage in databases, are more difficult to fully de-identify than quantitative data, and are at greater risk for improper analytical interpretation. However, these data are available from the corresponding author on reasonable request.

## Abbreviations

DART: Direct antigen rapid testing
COVID-19: coronavirus disease 2019
RT-PCR: Reverse transcription polymerase chain reaction
