# Supplementary material for: Caregiver perceptions of in-home COVID-19 testing for children with medical complexity: a qualitative study
Source: BMC Pediatr. 2022 Sep 8;22:533. doi: 10.1186/s12887-022-03550-5 (PMC9452877; doi:10.1186/s12887-022-03550-5)
Supplement: Supplementary file 1 — Additional file 1. Semi-Structured Interview Guide. [file 12887_2022_3550_MOESM1_ESM.docx]

Supplementary File

Semi-Structured Interview Guide

*First, let’s talk about the training that you received to do the testing at the start of the study.*

- Did the training prepare you to do the testing? (provide you the information you needed)
- What do you do differently now compared to what you were trained to do?
- Is there anything the study staff could have provided you that would have improved the testing experience or interpreting results -such as other supplies or handouts?

*We have a few questions to wrap up this section.*

- Have you ever had a positive test?
  - If yes, what did you do next?
  - If no, what would you do if a test came back positive?
- Have you ever done the test on another household member or yourself? How was that?
- Have you had any challenges performing the test? What did you do?
- Have you had any challenges interpreting the test? What did you do?
- Has your child had any negative experiences while having the test performed?
- Is there anything about the testing process that annoys, distresses, or bothers you?
- How would you rate your confidence while performing the test (1-10)? Ask them to explain why they feel this way.

*Next, I would like to ask a few questions regarding your child’s plans for back to school.*

- Does this testing process influence your confidence in having your child back to school in-person? Why or why not?

*We would now like to ask you some follow up questions.*

- Do you ever do anything to help your child feel more comfortable or cooperative while doing the test?
- In general, how do you feel about performing nasal swab testing at home? Are there benefits or drawbacks?
